# Strengthening primary health care resilience through community innovation: a qualitative case study from Quito’s response to COVID-19

**DOI:** 10.1186/s12939-025-02620-1

**Published:** 2025-10-10

**Authors:** Betzabé Tello, Iván Dueñas-Espín, Laura Di Giorgio

**Affiliations:** 1 CRECER Research Group (Cuidado Responsable, Equidad, Ciencia, Educación y Respeto), Pontificia Universidad Católica del Ecuador, Centro de Investigación para la Salud en América Latina (CISeAL), Quito, Ecuador; 2 CRECER Research Group (Cuidado Responsable, Equidad, Ciencia, Educación y Respeto), Pontificia Universidad Católica del Ecuador, Instituto de Salud Pública de la PUCE, Quito, Ecuador; 3 World Bank, Washington, DC USA

**Keywords:** Primary health care, Resilience, Community health teams, Health systems, Social determinants of health, COVID-19, Atención Primaria de Salud, Resiliencia, Equipos de salud comunitaria, Sistemas de salud, Determinantes sociales de la salud, COVID-19

## Abstract

**Background:**

This study aimed to analyse and document how Quito, the capital city of Ecuador, transformed its Primary Health Care (PHC) model following the COVID-19 pandemic. The reform involved a shift from a reactive, hospital-centred response to a community-based model focused on prevention, social determinants of health, and local engagement.

**Methods:**

This study used a qualitative case study design with a comparative and deductive approach, featuring 11 semi-structured interviews with health professionals, decision-makers, and administrative staff, alongside a review of secondary sources, including national and municipal legal framework (such as the Organic Code and the municipal Organic Statute), ordinances, the Municipal Code, and governance documents and accountability reports from three mayoral administrations. Thematic analysis identified key enabling factors and compared Quito’s experience with other cities in Ecuador and Latin America.

**Results:**

The central innovation was the creation of multidisciplinary Community Health Teams (Equipos de Salud Comunitaria, ESC) assigned to each of Quito’s 65 parishes. These teams focused on health promotion, disease prevention, and intersectoral coordination. Their implementation was supported by continuous training, integration of digital tools, community-based communication strategies, and protocols for operating in high-risk environments. A major driver of the reform was the political negotiation to sustain the increased health budget beyond the pandemic, enabling the long-term operation of ESCs. Ensuring the physical safety of health personnel also became a core component of the model, supported by risk mapping, emergency communication systems, and coordination with local security actors. The model was institutionalized through legal frameworks and aligned with national initiatives such as the Healthy Municipalities Programme and Comprehensive Family, Community and Intercultural Health Care Model (MAIS-FCI). The model demonstrated improved territorial access, community trust, and responsiveness amid rising urban violence, but faces limitations in technological infrastructure, monitoring indicators, and long-term sustainability.

**Conclusions:**

Quito’s experience highlights the potential of decentralised municipal governance to lead PHC reform through integrated, preventive, and community-based strategies. The findings provide valuable lessons for other cities in low- and middle-income countries seeking to enhance PHC resilience in fragile contexts. Strengthening digital capacity and ensuring institutional protection and funding for ESC will be key to sustaining progress.

## Resumen

**Antecedentes:**

Este estudio tuvo como objetivo analizar y documentar cómo Quito, la capital de Ecuador, transformó su modelo de Atención Primaria de Salud (APS) tras la pandemia de COVID-19. La reforma implicó un cambio de un enfoque reactivo y hospitalario hacia un modelo basado en la comunidad, centrado en la prevención, los determinantes sociales de la salud y la participación local.

**Métodos:**

Se utilizó un diseño cualitativo de estudio de caso, con un enfoque comparativo y deductivo, que incluyó 11 entrevistas semiestructuradas con profesionales de la salud, tomadores de decisión y personal administrativo. Además, se revisaron fuentes secundarias, como marcos legales nacionales y municipales (incluyendo el Código Orgánico y el Estatuto Orgánico municipal), ordenanzas, el Código Municipal y documentos de gobernanza e informes de rendición de cuentas de tres administraciones municipales. El análisis temático identificó factores facilitadores clave y comparó la experiencia de Quito con la de otras ciudades en Ecuador y América Latina.

**Resultados:**

La innovación central fue la creación de Equipos de Salud Comunitaria (ESC) multidisciplinarios, asignados territorialmente a las 65 parroquias de Quito. Estos equipos se enfocaron en la promoción de la salud, la prevención de enfermedades y la coordinación intersectorial. Un motor clave de la reforma fue la negociación política para mantener el presupuesto en salud incrementado más allá de la pandemia, lo que permitió la operación sostenida de los ESC. Garantizar la seguridad física del personal de salud también se convirtió en un componente esencial del modelo, apoyado por mapas de riesgo, sistemas de comunicación de emergencia y coordinación con actores locales de seguridad. El modelo fue institucionalizado mediante marcos legales y su alineación con iniciativas nacionales como el Programa de Municipios Saludables y el Modelo de Atención Integral de Salud Familiar, Comunitario e Intercultural (MAIS-FCI). Un motor clave de la reforma fue la negociación política para mantener el presupuesto en salud incrementado más allá de la pandemia, lo que permitió la operación sostenida de los ESC. Garantizar la seguridad física del personal de salud también se convirtió en un componente esencial del modelo, apoyado por mapas de riesgo, sistemas de comunicación de emergencia y coordinación con actores locales de seguridad.

**Conclusiones:**

La experiencia de Quito demuestra el potencial de la gobernanza municipal descentralizada para liderar reformas de APS mediante estrategias integradas, preventivas y basadas en la comunidad. Los hallazgos ofrecen lecciones valiosas para otras ciudades de países de ingresos bajos y medianos que buscan fortalecer la resiliencia de sus sistemas de APS en contextos frágiles. Fortalecer la capacidad digital y asegurar la protección institucional y el financiamiento de los ESC será clave para mantener los avances logrados.

## Introduction

The COVID-19 pandemic exposed critical vulnerabilities in health systems around the world [1]. In Ecuador, particularly in Quito, the capital, longstanding structural inequalities and a fragmented healthcare system exacerbated the challenges posed by the health crisis. Primary Health Care (PHC), despite being the foundation of the Ecuadorian health system, struggled to meet the needs of a rapidly evolving emergency [2]. However, amidst the COVID-19 crisis, Quito managed to pivot and innovate, transforming its traditionally reactive PHC system towards a community-based, preventive, and resilient model.

PHC is globally recognised as a fundamental strategy for achieving universal health coverage, promoting equity and ensuring health system sustainability [3]. Rooted in the Alma-Ata Declaration and reaffirmed in Astana, it embodies the principles of accessibility, prevention, community participation and, intersectoral action [3]. In crisis contexts, PHC becomes a cornerstone of resilience, defined as the capacity of health systems to absorb shocks, adapt and transform while maintaining essential functions [4].

Resilient PHC systems are characterised by local responsiveness, continuity of care and trusted relationships between providers and communities [5]. These attributes enable early detection of health threats, preserve service delivery under pressure and facilitate recovery [5]. The World Health Organization’s six building blocks of health systems are service delivery, health workforce, information systems, essential medicines, financing, and leadership [6]. These components are especially relevant to efforts that strengthen resilience and enable effective coordination across sectors and levels of care [6].

However, achieving resilience requires more than strong system design [7]. It involves institutional adaptability, legal frameworks that support decentralised decision-making and inclusive governance mechanisms [8]. Global and regional evidence shows that robust PHC platforms, those that integrate social determinants, intersectoral action, and trusted community mechanisms, can maintain essential services and recover more effectively in times of crisis [9]. In contrast, fragmented systems with weak first-level care structures struggled to reach vulnerable populations and manage community-level transmission [9].

The pandemic highlighted the urgen need to reconfigure PHC systems with resilience at the centre, particularly in low- and middle-income countries (LMICs) where the capacity to absorb shocks and maintain essential services is often limited [6]. Community-based interventions are especially crucial in fragile or conflict-affected settings, where descentralised and participatory approaches can prevent service disruption, protect vulnerable populations and, foster trust in institutions [6].

Quito’s experience contributes to this global imperative, offering a locally adapted, community-centred strategy that prioritised preventions, territorial governance and institutional innovation [10]. The city’s approach draws from multiple lessons: the importance of health worker protection in insecure settings, integration of digital technologies, community engagement, and sustained political will [10].

**Fig. 1:**
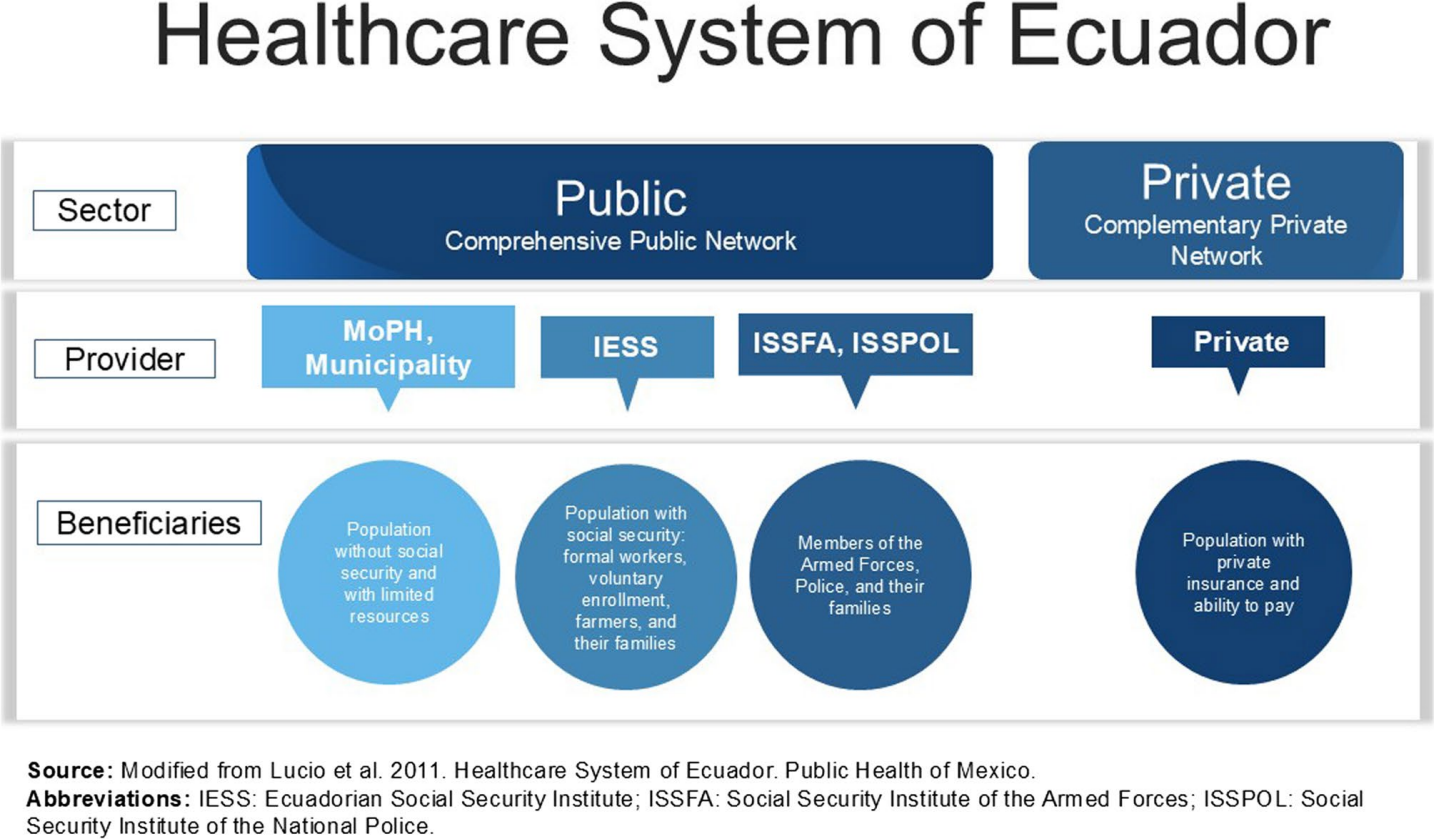
Structure of the Ecuadorian health system

To understand how this transformation occurred, it is essential to examine the national and local policy frameworks. Ecuador’s health system is a mixed model composed of public and private providers, with the Ministry of Public Health (MoPH) as the main public authority responsible for stewardship and service provision [11]. Other public subsystems such as the Ecuadorian Social Security Institute (IESS), the health services of the armed forces and police, and local governments with decentralised health responsibilities, operate in parallel. These institutional arrangements function independently with limited coordination, contributing to the overall fragmentation of the health system [12] (Fig. 1).

**Fig. 2:**
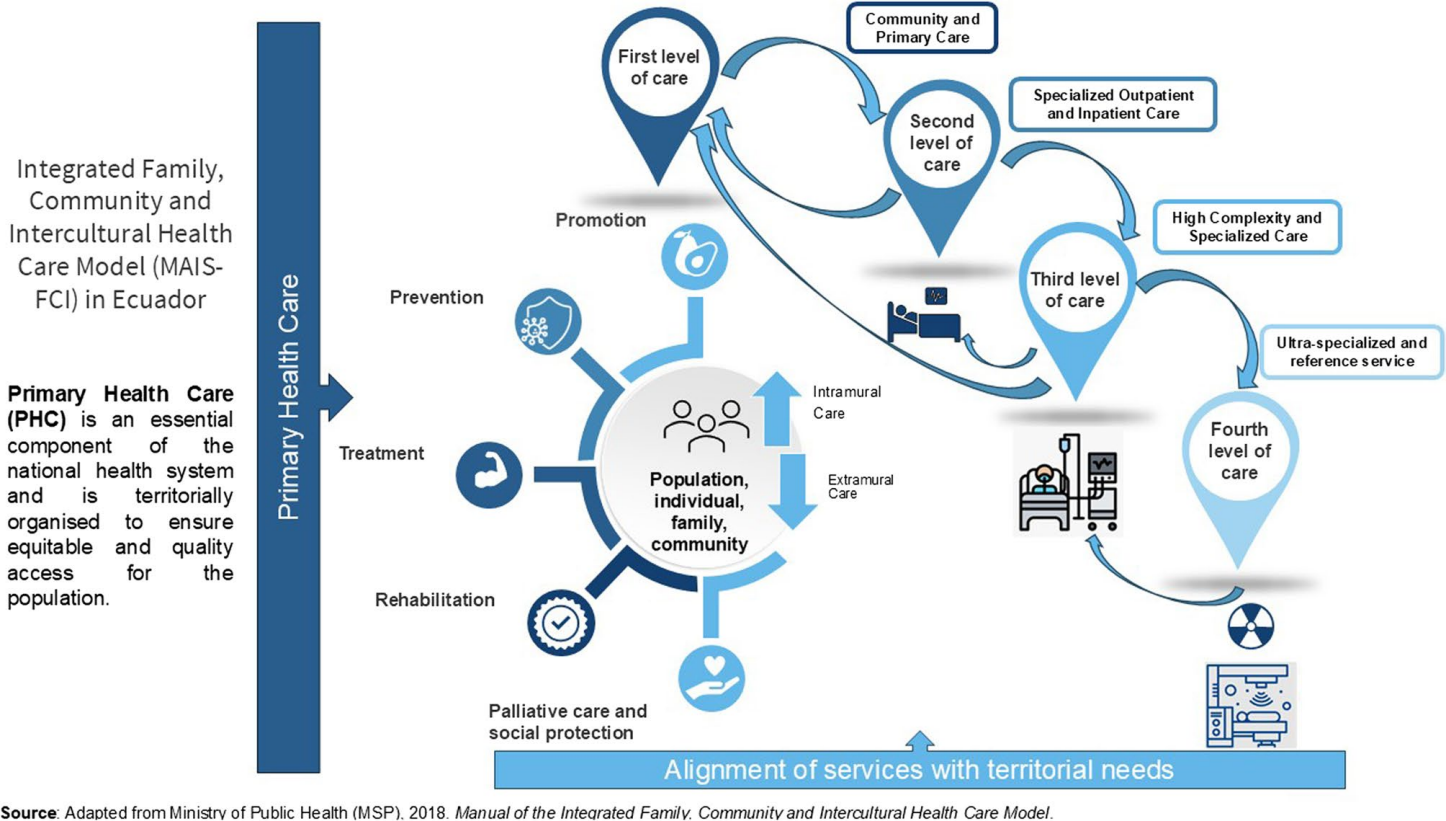
MAIS-FCI: comprehensive family, community and intercultural health care model

The national PHC policy is based on the Modelo de Atención Integral de Salud Familiar, Comunitario e Intercultural (MAIS-FCI), which emphasises integrated care, intersectoral coordination, and cultural responsiveness. However, implementation has been uneven across territories [11] (Fig. 2).

In this context, the Metropolitan District of Quito (Distrito Metropolitano de Quito, DMQ) developed a complementary municipal strategy aligned with national priorities but tailored to local governance structures and territorial deployment. Government by Autonomous Descentralised Government (Gobierno Autónomo Descentralizado, GAD), Quito’s health system includes a dedicated Health Secretariat (Secretaría de Salud), responsible for local planning, coordination and service delivery at the community level [13].

Following the pandemic, the municipality initiated a transformation of its PHC model, shifting from reactive, episodic care to a territorial, community-based strategy. This study analyses transformations and offers insights on how local governments can institutionalise resilient PHC models through legal, financial and multisectoral mechanisms. Specifically, this study seeks to answer the following research question: How did the Municipality of Quito transform and institutionalise a resilient, community-based Primary Health Care model during and after the COVID-19 pandemic?

## Methods

### Study design

A qualitative approach was applied for this study to enable in-depth analysis of policy decisions, governance processes, and operational strategies that shaped the emergence and institutionalisation of the new PHC model. The methodology was primarily exploratory and interpretive, relying on the perspectives of key informants and policy actors involved in the design and implementation of the municipal response.

### Aim

This study aimed to analyse and document how the Metropolitan District of Quito, Ecuador, transformed its PHC model in response to the COVID-19 pandemic. Specifically, it examined the shift from a reactive to a community-based and preventive approach, identified key enablers of this transformation, and explored the strategies used to institutionalise the new model within municipal health structures.

### Setting

The study was conducted in Quito, Ecuador’s capital and the second most populous city in the country, with nearly three million inhabitants. The city is governed by an Autonomous Decentralised Government (Gobierno Autónomo Descentralizado, GAD), which maintains a dedicated Health Secretariat (Secretaría de Salud) responsible for implementing municipal-level public health strategies.

The municipality’s health mandate is focused on disease prevention and health promotion, particularly among groups under municipal responsibility and priority populations. These include young children, older adults, people with disabilities, migrants and refugees, informal workers (such as vendors and recyclers), sex workers, LGBTIQ + populations, people who use psychoactive substances, children exposed to child labor, people living on the street, and groups historically excluded from the health system. The PHC system in Quito operates in integrated within the broader public health framework but operates under decentralised municipal management, with a focus on health prevention, disease prevention, concurrent care, rehabilitation, intersectoral action, and local engagement. The transformation analysed in this study was led by the municipal authorities between 2014 and 2024.

### Data collection

Data were collected between July and September 2024 through two primary sources: semi-structured interviews and documents review. A total of 11 interviews were conducted with purposively selected participants, including municipal health officials, administrative staff, decision-makers, and frontline public health professionals such as physicians, nutritionists, and nurses involved in the implementation of the reform process. Participants were recruited based on their roles in planning, implementing or evaluating the new PHC model. Interviews followed a flexible thematic guide and were conducted in Spanish, lasting between 45 and 90 min. All interviews were recorded, transcribed verbatim, and anonymised before analysis.

In addition to interviews, we reviewed documents such as municipal ordinances and resolutions, strategic planning documents, operational protocols, budgetary reports, and public health programme evaluations issued by the municipality between 2014 and 2024. These documents provided contextual information and were used to triangulate and corroborate findings from the interviews.

### Data coding and analysis

We conducted a thematic analysis using a combination of deductive and inductive coding. Deductive coding was guided by a predefined analytical framework based on the WHO health system building blocks and literature on PHC resilience and institutional transformation [14, 15]. In parallel, inductive coding allowed for the identification of emergent themes grounded in the participants’ narratives and document content, without prior assumptions.

All interviews and documents were analysed in Spanish, the language in which data were originally collected. Coding was performed using ATLAS.ti qualitative analysis software. Two researchers independently applied initial codes, which were then reviewed and refined jointly to ensure consistency and coherence in the analytical categories.

An initial coding matrix was developed around key thematic areas such as governance, resilience, PHC models, and institutional change. Final themes were organised across three analytical levels:

1. Governance and institutional mechanisms.
2. Community-based operational strategies.
3. Enabling conditions for sustainability and resilience.

In addition to the thematic analysis of Quito’s experience, we incorporated a comparative analysis. Key findings were contrasted with documented PHC responses in other Latin American and global urban contexts, particularly during the COVID-19 pandemic. This step drew on peer-reviewed literature, institutional reports, and case studies to identify transferable lessons and context-specific enablers of PHC resilience.

**Fig. 3:**
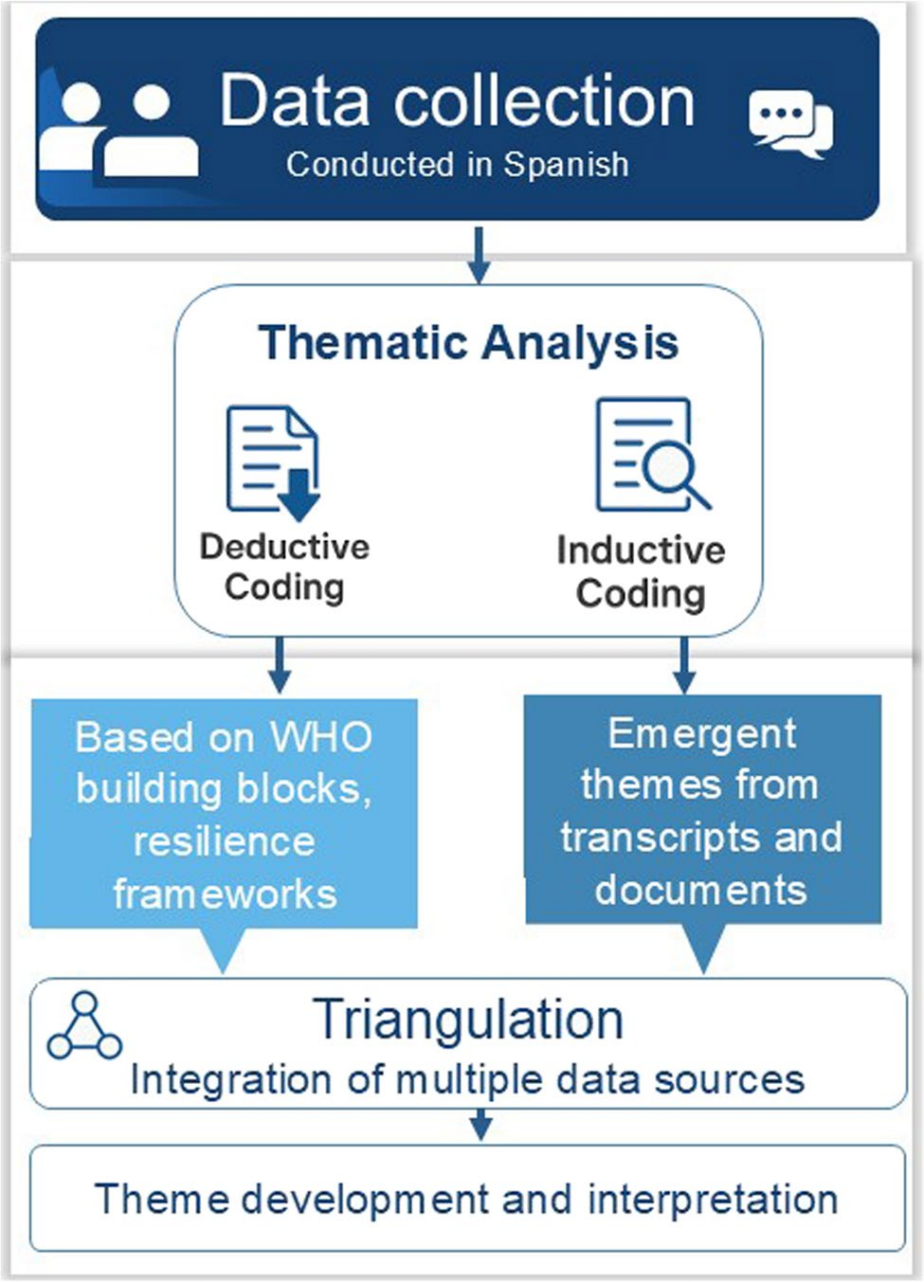
The qualitative analysis process

Triangulation of interviews, official documents, and external case examples helped enhance the validity and depth of the analysis. Select quotations were translated into English after analysis for inclusion in the manuscript. (Fig. 3)

## Results

### Innovative strategies in the PHC model during the pandemic

The interviews with frontline health professionals in Quito revealed a significant transformation of the municipal health model during and in the aftermath of the COVID-19 pandemic, particularly in relation to the evolution of Community Health Teams (CHT). This shift involved the adoption of a community-based preventive approach that integrated social determinants of health and prioritised local engagement and territorial planning. The transformation was supported by political, financial and legal efforts, including the development of frameworks to ensure accountability and sustainability through monitoring and evaluation.

**Fig. 4:**
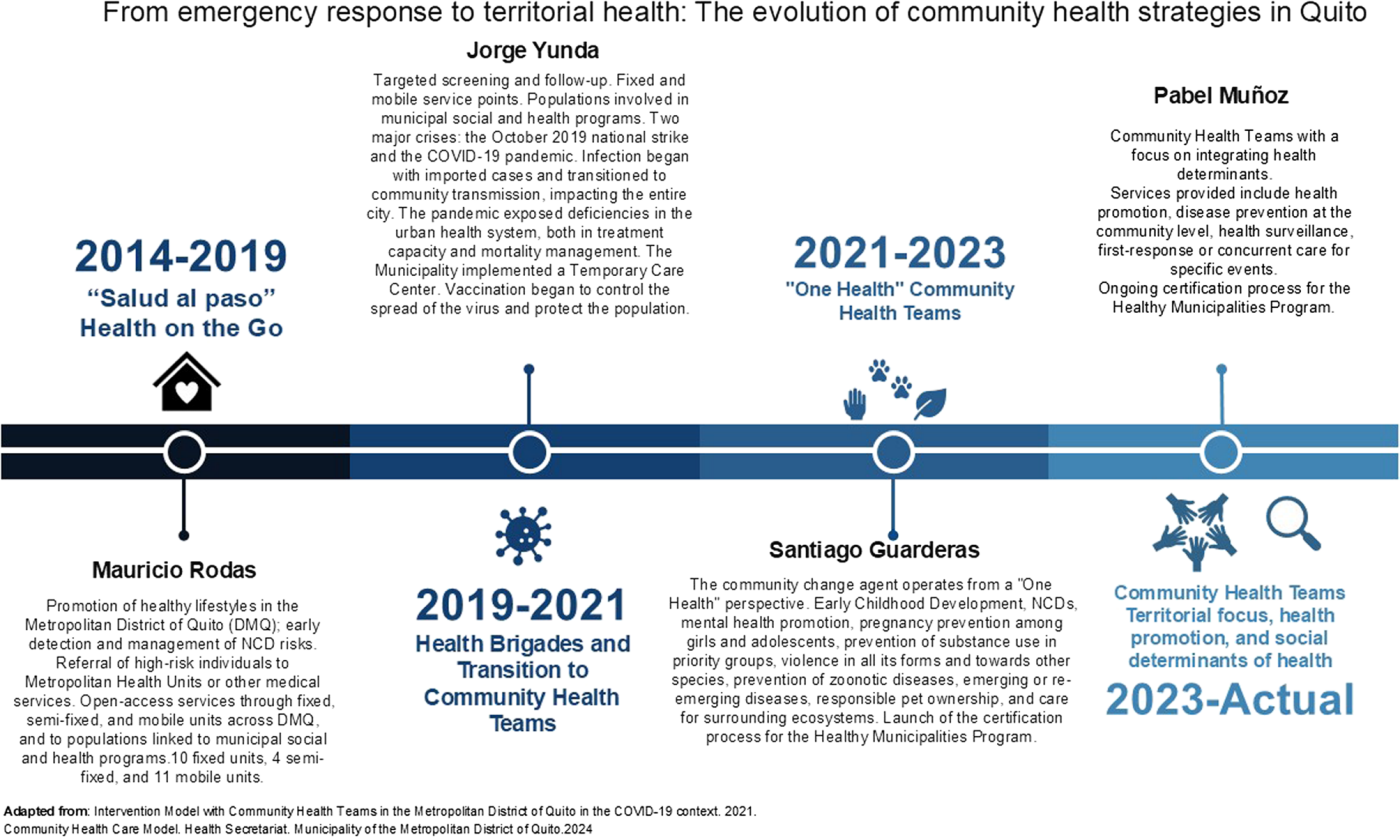
Evolution of Quito’s community health strategy during and after the COVID-19 pandemic

A key component of the reform was the evolution of the CHT (Equipos de Salud Comunitaria, ESC), which emerged from COVID-19 response brigades and assumed broader preventive and promotional responsibilities. The roles of health professionals, including physicians, nutritionists and nurses, were expanded to include intersectoral coordination, health education and community-level action. This shift required significant adaptation, including training in new protocols, enhanced use of digital tools and community-based safety measures in high-risk environments (Fig. 4).

Professionals described an increased use of social media and virtual platforms to deliver workshops and health education, in response to both user preferences and pandemic restrictions. Home visits were highlighted as an effective tool for identifying household-level nutrition risks and addressing them through personalised counselling.

*“Now many of our workshops are delivered on Zoom because it’s more comfortable for the user*,*”* explained a nutritionist.

The adoption of digital tools and the adaptation of health protocols were accelerated during the pandemic, enabling the continuity of community-based health services. Examples include changes in biosecurity, vaccine administration, data recording and risk management procedures. The reform also prompted a shift in institutional thinking, with growing emphasis on preventive care and population-level health indicators rather than disease treatment alone.

Health professionals and former municipal officials noted that public investment increased significantly during the health emergency. To maintain these resources post-pandemic, officials strategically reframed the budget to support permanent CHT and aligned them territorially by parish. This transition from individual-level nutrition care to community-centred prevention was seen as essential to address emerging public health needs.

“*We no longer focus only on treating COVID-19 cases but on the consequences of the pandemic. We even reached 95–98% vaccination coverage. Our teams became experts and began supporting the national immunisation programme*,*”* a former health official explained.

This transformation laid the foundation for a more resilient and territorially responsive PHC system, repositioning CHT as long-term agents of health promotion, prevention, and intersectoral action in Quito.

### Resilience factors

Quito’s health response demonstrated significant resilience capacities at both the institutional and community levels. Community resilience was strengthened through the formal deployment of the CHT (Equipos de Salud Comunitaria, ESC), the use of local knowledge, and the integration of health professionals into community dynamics. Teams adapted protocols, redefined roles and built stronger relationships with residents, fostering trust and responsiveness.

A central driver of this resilience was continuous training. Health professionals were regularly updated on operational protocols, digital tools, risk communication, and intersectoral collaboration. Training sessions helped staff adapt to the evolving demands of the post-pandemic context and reinforced their capacity to act beyond clinical care.

Importantly, financial autonomy enabled by Quito’s decentralised governance structure allowed the municipality to rapidly mobilise and sustain health resources. During the pandemic, the health budget expanded significantly. After the emergency, municipal leaders defended the continued investment by reframing the crisis in terms of persistent public health challenges, such as food insecurity, mental health, and the long-term impacts of COVID-19.

*“How do we justify the budget if there is no longer an emergency? There may be no emergency*,* but there is a public health problem… Otherwise*,* we go back to what we had before*,*”* noted a former municipal official.

*“To maintain the budget*,* we pivoted to a community health model. One team per parish allowed us to optimise resources*,* retain funding*,* and ensure territorial organisation*,*”* the same official added.

Occupational safety and psychosocial support were also identified as resilience factors. In areas with high levels of violence or instability, CHT applied risk protocols and used digital systems to monitor team movements and limit exposure.

*“There’s a group chat where everyone checks in and out… if there’s no safety guarantee*,* we don’t send teams*,*”* explained a municipal officer.

Finally, the formal recognition of health workers by municipal authorities was essential to maintaining morale and motivation. Acknowledging the efforts of frontline staff reinforced their identity as agents of social transformation and built internal support for the reform.

### Institutionalisation of the new PHC model in Quito

The institutionalisation of Quito’s PHC model required a series of formal and strategic actions to secure its continuity beyond the pandemic. The enactment of legal frameworks was central to this effort, including Resolution AQ 025-2022, which enabled the implementation of the Healthy Municipalities Programme and provided a structure for measuring health indicators in areas such as environment, mobility and local economy. Resolution ADMQ 007-2024 formally updated the mandate of the Health Secretariat within the Organic Statute of the municipal government, clarifying its role in leading the strategy.

The creation of municipal ordinances and integration with national programmes helped consolidate a legal and operational foundation for the model. However, further development of the normative framework and secure resource allocation were identified as essential for long-term sustainability. Monitoring was seen as a critical mechanism to ensure accountability and to demonstrate the model’s impact. Both qualitative and quantitative indicators were used to evaluate performance, although the need for improved data systems and common metrics was frequently mentioned.

*“We now work with indicators. These are what will help us measure our common impact*,*”* stated one municipal administrator.

*“We have the Metropolitan Development and Land Use Plan and the National Healthy Municipalities Programme. This programme includes indicators to address social determinants*,” explained an administrative official when asked about the tools used to anchor the strategy.

The institutional framework was supported by a network of intersectoral coordination mechanisms, most notably the creation of Zonal Intersectoral Committees. These bodies facilitated collaboration among municipal agencies, enabling joint responses to community health determinants such as environmental risk and informal alcohol sales. Municipal agencies including the Environment Secretariat became active participants in addressing upstream determinants of health through targeted interventions.

*“We are proposing something that did not exist before in the municipality… Zonal Intersectoral Committees operating from the zonal administration. The significant increase in the health budget during the pandemic made it possible to improve resource management*,” stated a decision-maker.

Regarding the role of municipal agencies, coordinated work was highlighted as a model of collaborative governance.

*“For example*,* the Environment Secretariat provides responses that are grounded in the practice of addressing health determinants*,” explained another decision-maker.

Community participation became a key pillar of institutionalisation, enabling local demands to shape public policy. CHT worked not only on health issues but also on broader public concerns, such as regulating alcohol in unsafe spaces. This reflected stronger coordination with municipal governance, which, as a local government, integrates agencies, public companies, and secretariats to address social determinants of health, facilitating intersectoral collaboration and policy coherence.

*“It’s no longer about telling people not to drink. Now we work with the municipality to shut down illegal bars*,*”* one decision-maker explained.

Challenges identified included cultural differences between parishes, community reluctance to shift from curative to preventive services and resistance from some health units still focused on traditional service delivery.

The process of institutionalising the PHC model in Quito demonstrated the importance of resilience, multisectoral governance and bottom-up adaptation. It highlighted how health systems can transition from emergency response to long-term transformation when legal frameworks, community engagement and political will align.

*“Unit directors still think that teams need to provide direct care… as if that’s what gives them legitimacy*,* even if the care is of poor quality. But these teams no longer respond to a traditional health service logic; they respond to needs emerging from municipal management”*, stated a decision-maker.

**Fig. 5:**
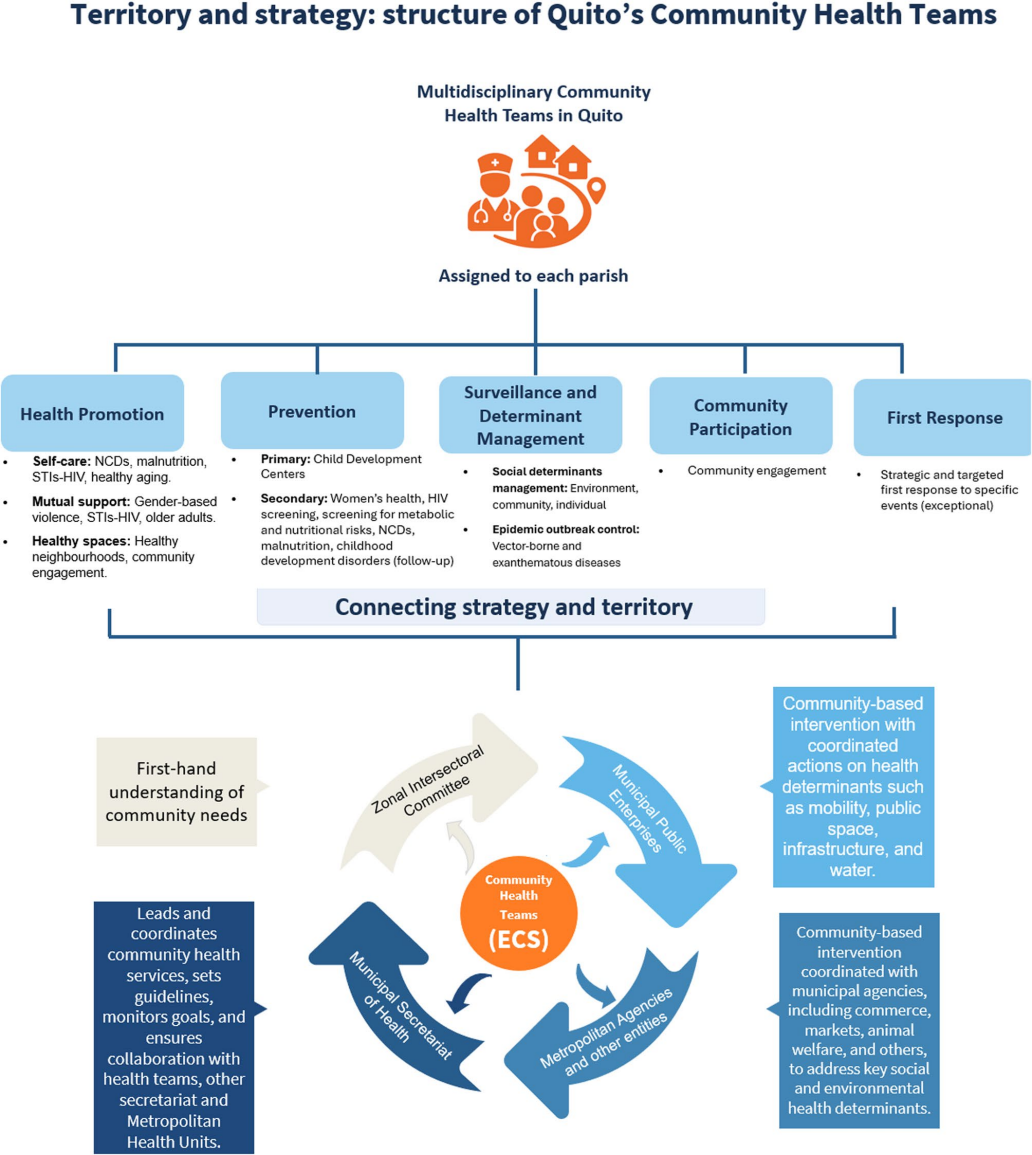
Current community-based primary health care model in Quito

The institutionalisation of Quito’s new PHC model demonstrates that transforming health systems requires more than technical adjustments; it demands political commitment, territorial governance, and the ability to embed health strategies within broader development frameworks. While important legal and operational mechanisms have been established, concerns remain about its long-term sustainability, the need for a robust monitoring strategy, and the importance of institutional protection for community-based strategies in politically dynamic contexts. Sustaining this model will depend on continued intersectoral collaboration, long-term financing, and the consolidation of monitoring systems that can demonstrate impact and legitimacy (Fig. 5).

## Discussion

This study explored transforming and institutionalising a new community-based PHC model in the Metropolitan District of Quito during and after the COVID-19 pandemic. The findings revealed how local governments can reposition health systems to better address public health challenges through territorial governance in a LMIC can reorient its health system through territorial governance, intersectoral collaboration, and community participation to strengthen resilience and promote equity.

One of the key achievements in Quito was the role of municipal leadership in institutionalizing the new model through legal frameworks, dedicated budgets, and administrative continuity. The creation of Zonal Intersectoral Committees facilitated cross-sector coordination and enabled health to be integrated into broader social agendas. These structures align with the World Health Organization’s emphasis on governance and leadership as essential pillars of resilient health systems and reflect recent frameworks that highlight institutional legitimacy and political commitment [14, 15]. The ability to sustain increased health budgets after the emergency phase was essential to ensuring the ongoing operation of the CHTs (Equipos de Salud Comunitaria, ESC), setting a precedent for fiscal sustainability in decentralized systems.

The shift from reactive, episodic model to one based on prevention, and community engagement strengthened the city’s capacity for early case detection, epidemiological surveillance, and timely response [16]. Assigning one CHT per parish enabled tailored interventions at the local level [17]. Transparent communication and the involvement of community leaders helped build trust and overcome resistance to the new model. These elements reinforce the importance of proximity, local responsiveness, and participatory approaches as key enablers of system resilience [18].

Community engagement in Quito’s PHC model was operationalized through a structured territorial approach that placed Community Health Teams at the center of local-level interaction. Each team was assigned to a specific parish and was responsible for conducting regular neighborhood visits, establishing relationships with local leaders, and coordinating health promotion and disease prevention activities alongside existing community committees. Far from being passive recipients of care, community members participated in participatory planning processes, including needs assessments, risk mapping, and prioritization of outreach strategies [19]. This engagement was institutionalized through the role of CHTs as intermediaries, who documented community concerns and relayed them to municipal health authorities to inform service planning and decision-making. While formal representation mechanisms were still evolving, this dynamic interface enabled a continuous feedback loop and helped align the municipal health response with community needs [20].

Although COVID-19 vaccination was not the primary focus of this study, available data highlight the responsiveness of the municipal health system. By the end of 2021, over 86% of residents aged three and older in the Metropolitan District of Quito had received the full vaccination schedule, comparable to or even higher than the national average, particularly in Pichincha [21, 22]. These results reflect both strong national-local coordination and the strengthened PHC model’s ability to implement large-scale public health interventions during emergencies [6, 23].

The implementation of the new model also required rapid reorganization of the health workforce. Professionals were trained to take on new roles in prevention, digital service delivery, and intersectoral coordination. This organizational flexibility reflects an adaptive learning capacity essential for navigating uncertainty [20, 24]. However, challenges remain regarding the availability of personnel, particularly in peripheral or underserved parishes.

The successful implementation of the community-based PHC model in Quito was also supported by a series of targeted training activities for health personnel, especially members of the Community Health Teams. These trainings covered key competencies for territorial work, including social determinants of health, risk management, nutrition, mental health, educommunication strategies, prevention of harmful substance use, and the strengthening of community teams themselves. Although there was no single standardized program solely focused on community engagement techniques, key principles of communication and participation were embedded across all modules [25, 26]. Additionally, professionals were trained to coordinate health promotion and disease prevention actions with local leaders and institutions [19]. These capacity-building efforts were essential not only for the rapid adaptation of roles during the pandemic but also for sustaining the long-term institutional transformation of the local health system [5, 27, 28].

In terms of technology, Quito implemented teleconsultation platforms, virtual mental health services, and digital tools such as KoboToolbox for community activity monitoring. These initiatives marked important progress toward digitalizing key components of municipal health services and facilitated continuity of care during mobility restrictions. However, when comparing the case of Quito with other urban health systems, caution must be exercised in overstating the role of advanced technologies such as artificial intelligence or real-time surveillance tools [29]. While such innovations have been implemented in some metropolitan areas to optimize logistics, support early outbreak detection, and inform public health responses, their relevance and effectiveness remain highly context-dependent, particularly in community-oriented PHC settings, where standalone technological solutions have shown limited empirical impact [30, 31]. In fact, recent syntheses indicate that health system resilience in LMIC has been more consistently associated with decentralized governance, sustained community engagement, and strengthened operational capacity than with high-end technological inputs [30]. For example, during the refugee crisis in Lebanon, health system resilience was preserved not through digital sophistication but through networked governance, diversified financing, and inclusive service delivery mechanisms [32].

Consequently, any recommendation to incorporate specific technologies into the municipal health system of Quito should be grounded in contextually relevant evidence of impact from cities with similar institutional, financial, and infrastructural constraints. Without such evidence, there is a risk of adopting interventions whose benefits may be marginal or even misaligned with the needs of the most vulnerable populations. In Quito, digital platforms were primarily used for basic triage, remote consultation, and communication with the population, yet their long-term utility remains limited by digital literacy gaps and inequitable access to connectivity. Therefore, rather than advocating for generalized deployment of artificial intelligence or predictive analytics, future efforts should prioritize the development of interoperable health information systems, networks of community health workers, and simple digital tools that reinforce, but do not replace, territorial and relational engagement [14, 31]. This is consistent with comparative experiences in cities like Bogotá and Lima, where the resilience of PHC was anchored in the expansion of local teams, interinstitutional coordination, and trust-based community mechanisms, rather than technological sophistication [33]. Frameworks for assessing urban health system resilience should therefore emphasize not only infrastructure and data systems but also institutional legitimacy, adaptability, and participatory governance, dimensions that have gained increasing relevance in the literature on resilient health systems. According to the resilience framework proposed by Paschoalotto et al. (2023), several components are considered critical for strengthening health systems, particularly in crisis response contexts. This framework emphasizes governance and leadership as foundational pillars, alongside the integration of communication and social participation as essential elements that connect system performance with broader contextual realities. Moreover, the role of information systems and digital infrastructure is acknowledged for their potential to support coordination and evidence-informed decision-making [14].

A distinctive feature of Quito’s approach was the municipality’s ability to maintain increased health funding beyond the acute phase of the pandemic. While many jurisdictions scaled back post-emergency investments, Quito reframed health spending as a structural necessity, given the city’s high levels of social vulnerability and historical underinvestment. This long-term financial commitment was vital to institutionalizing the CHT model and underscores the importance of local fiscal autonomy in building a resilient health system [25].

Security in urban neighborhoods with high levels of violence also posed significant operational challenges [34]. The municipality responded with emergency protocols, designated safe service points, and coordination with security forces. The protection of health personnel is widely recognized as a key determinant of service continuity, particularly in fragile or conflict-affected contexts. Institutional resilience strategies must therefore include physical and emotional safety measures for healthcare workers [18, 35].

Compared to international experiences, Quito’s model stands out for its institutional innovation, territorial approach, and strong community engagement. However, key gaps remain, particularly in digital infrastructure, results-based monitoring, and technological self-sufficiency. Strengthening evaluation systems, developing interoperable information platforms, and building sustainable strategies for workforce training and protection will be essential for advancing health system resilience [15].

Table 1 offers a comparative overview of Quito’s COVID-19 response in relation to other international cities. While Quito implemented essential digital tools such as teleconsultations and a basic mobile health app, cities like Mexico City, Bogotá, and Lima adopted more advanced technologies, including artificial intelligence for symptom tracking and real-time data dashboards [36, 37]. Regarding service delivery, Quito mobilized community testing brigades and triage tents, and later established permanent Metropolitan Health Units and 65 Community Health Teams, one per parish, to enhance territorial reach [38]. In contrast, other cities deployed large-scale field hospitals [39]. The table also underscores Quito’s effective community participation model, where local leaders and volunteers played key roles in monitoring and outreach, now institutionalized through Zonal Intersectoral Committees. Finally, it highlights how Quito collaborated with national and international partners during the pandemic, partnerships that have since been formalized to support routine services and emergency preparedness [40].

**Table 1:** Comparison of quito’s pandemic response with international experiences

Dimension	Quito – Pandemic (2020–2021)	International Cities	Quito – Current (2022–2025)
Technological Innovation	Teleconsultations, mobile apps (171 line, CNEA, MSP appointment platforms); limited community WhatsApp use	AI-enabled apps, georeferenced monitoring, digital self-assessment tools (Mexico City, Bogotá, Madrid, Lima)	Social media strategies, virtual health education workshops, expanded digital community engagement
Health Facility Capacity	Temporary triage units, mobile brigades, converted schools and sports venues	Rapid ICU expansion, field hospitals, modular clinics (Barcelona, Bogotá, Lima)	Three metropolitan health centers (CMIs); one Community Health Team (CHT) per parish (strengthened territorial model)
Community Integration	High involvement of parish leaders, local brigades, and community kitchens	More centralized approaches in some cities; exceptions include Bogotá’s Local Action Plans	Active territorial engagement by CHTs; incorporation of community assemblies and safe space promotion
Intersectoral Coordination	Collaboration among MoPH, Quito Municipality, UN agencies, academia	Multi-agency platforms (e.g., Spain’s Interterritorial Council, Mexico’s Interinstitutional COVID Cabinet)	Zonal Intersectoral Committees (CZIs) and Community Technical Boards; co-management of health and social services
Resource Mobilization	Emergency hiring, food kits, PPE delivery	Large-scale medical equipment logistics, donation channels (e.g., Bogotá, Lima), R&D financing (Madrid, CDMX)	Sustained municipal budget for public health; strategic CHT deployment and stable workforce contracts
Digital Health	Psychological helplines, phone-based triage	Health monitoring apps, home-based telemedicine (Barcelona, CDMX)	Virtual outreach, digital literacy campaigns, integration of Kobo Toolbox for field data
Logistics and IT Systems	Major gaps in data integration, poor coordination at early stages	Real-time dashboards, traceability of supply chains (Madrid, Bogotá)	Use of Kobo Toolbox, mobile tablets for CHTs; data flows improved but interinstitutional integration remains partial
Community Services	Local food delivery, psychological first aid, community pharmacies	Centralized or public-private delivery in larger cities	Creation of “Safe Spaces for Youth” and gender-sensitive services coordinated with parishes and community actors
Vaccination Strategy	Coordination between MoPH and Municipal Emergency Operations Committee; use of public schools and parks	Use of digital vaccination registries, QR-based access control (Madrid, CDMX); university-led vaccine trials (Barcelona, Bogotá)	Continued coordination with MoPH; mobile teams for school-based and house-to-house immunization; reduced public demand since 2023

*CMI* Centro Metropolitano de Salud, *CHT* Community Health Team, *MoPH* Ministry of Public Health, *UN* United Nations, *CZIs* Zonal Intersectoral Committees, *PPE* Personal Protective Equipment, *CDMX * Ciudad de México, *IT* Information Technology

Despite important progress, several limitations persist. First, Quito’s digital infrastructure remains basic compared to other cities using AI and real-time surveillance. Strengthening technological capacity is essential for future preparedness, though recent tools like teleconsultation and KoboToolbox mark initial progress [6].

Second, although a range of administrative and service-level indicators are available, monitoring remains primarily focused on outputs, such as the number of services delivered, rather than on health outcomes, such as improvements in nutritional status or mental health. This output-oriented approach limits the ability to evaluate the true effectiveness and long-term impact of the PHC model, underscoring the need for a more comprehensive, outcome-based evaluation framework [9]. Although this study primarily relied on qualitative data, the municipal health strategy was accompanied by administrative monitoring systems that incorporated both quantitative and qualitative indicators. These tracked components such as the number of Community Health Teams (CHTs) deployed per parish and the volume of services provided. For example, municipal accountability reports indicate that 231,870 individuals received services through CHTs and municipal patronatos; 9,498 adolescents participated in sexual and reproductive health promotion workshops; 13,258 individuals accessed mental health services at the La Ronda Ambulatory Center; 34,602 students were reached through the Healthy Schools Strategy; and over 5,000 children under five received nutrition-related support through *Quito Wawas* (municipal early childhood care centers) and CEMEIs (Municipal Early Childhood Education Centers). While these figures do not emerge from an integrated monitoring and evaluation system, they offer valuable benchmarks to assess implementation progress and identify priorities for future system strengthening [25].

Third, tensions between curative expectations and the preventive community model highlight the need for continued institutional learning [41]. Fourth, ensuring health worker safety in violent urban areas remains a challenge despite existing protocols [42]. Finally, trust-building through local leadership and coordination with the Ministry of Health was key to sustaining service delivery [43].

One of the main strengths of this study is its in-depth exploration of a real-time institutional transformation process led by a local government in a LMIC context. The use of a qualitative design allowed for a rich understanding of the implementation dynamics, including political, administrative, and community perspectives that are often underrepresented in health systems research.

The triangulation of data sources, semi-structured interviews with diverse stakeholders, policy documents, and municipal planning instruments, enhanced the credibility and contextual depth of the findings. Additionally, the study provides valuable insights on how a decentralised PHC strategy can be institutionalised through legal frameworks, community structures, and intersectoral mechanisms.

By situating Quito’s experience within national and international comparisons, the study also contributes to the broader literature on urban health resilience, offering transferrable lessons for other cities seeking to strengthen their PHC models post-crisis.

This study also has limitations. First, it relied solely on qualitative data and did not include quantitative indicators to assess the health outcomes or service coverage changes resulting from the new PHC model. Although interviews and document review provided insights into the perceived impacts, the absence of routine data limited the ability to evaluate the model’s effectiveness empirically.

Second, the scope of the analysis was limited to the Metropolitan District of Quito, and findings may not be generalisable to other cities or rural settings in Ecuador. However, the analytical framework may be useful for examining similar processes in other decentralised contexts.

Lastly, some perspectives, particularly those of community members and users of health services, were not directly captured in the data collection process. Future research should incorporate these voices to assess the legitimacy, accessibility and perceived quality of services under the community-based model.

## Conclusion

The transformation of the PHC model in Quito illustrates the capacity of local governments to lead meaningful health system reforms through a community-based, preventive, and territorially structured approach. By institutionalising multisectoral coordination, fostering community engagement, and sustaining investments beyond the acute phase of the COVID-19 pandemic, Quito strengthened the resilience and responsiveness of its municipal health system.

Key innovations included the deployment of CHT (Equipos de Salud Comunitaria, ESC) at the parish level, the establishment of Zonal Intersectoral Committees to support inter-agency collaboration, and the alignment of health services with social determinants through legal and planning instruments. These strategies enabled the city to move beyond reactive crisis management and integrate health into broader local governance and development agendas.

Nonetheless, Quito’s experience also revealed persistent challenges. Technological infrastructure remains limited, and existing indicators focus primarily on service delivery rather than health outcomes, making it difficult to assess the model’s true impact. The lack of a comprehensive monitoring and evaluation framework, along with institutional resistance to shifting from curative to preventive approaches, continue to pose significant barriers to full implementation. Moreover, the need to ensure the physical safety of health personnel working in high-risk urban areas emerged as a core operational and ethical imperative. Ensuring protective mechanisms, such as risk-mapping, communication systems, and intersectoral coordination, must be viewed as essential to equitable and sustainable health service delivery.

As urban areas across LMIC seek to strengthen PHC through decentralised, community-driven approaches, Quito’s experience provides actionable insights. Future efforts should prioritise investment in digital health capacities, consolidation of monitoring and evaluation systems, protection for frontline health workers, and stable political and financial support to sustain innovations and embed them in resilient health systems beyond emergency contexts.

## Data Availability

No datasets were generated or analysed during the current study.

## Abbreviations

PHC: Primary Health Care
CHT: Community Health Teams
DMQ: Metropolitan District of Quito
LMIC: Low and Middle-Income Countries
MoPH: Ministry of Public Health
MAIS-FCI: Comprehensive Family, Community, and Intercultural Health Care Model
COVID-19: Coronavirus Disease 2019
ESC: Spanish acronym used for ESC Equipos de Salud Comunitaria; avoid duplication if only English abbreviation is used throughout CHT
